# Promising Agromaterials Based on Biodegradable Polymers: Polylactide and Poly-3-Hydroxybutyrate

**DOI:** 10.3390/polym15041029

**Published:** 2023-02-18

**Authors:** Yulia Victorovna Tertyshnaya, Maria Victorovna Podzorova, Ivetta Aramovna Varyan, Victor Victorovich Tcherdyntsev, Mikhail Yurievich Zadorozhnyy, Elena Valerievna Medvedeva

**Affiliations:** 1 Emanuel Institute of Biochemical Physics, Russian Academy of Sciences, 4 Kosygina Str., Moscow 119334, Russia; 2 Laboratory Advanced Composite Materials and Technologies, Plekhanov Russian University of Economics, 36 Stremyanny per., Moscow 117997, Russia; 3 Federal Research Agro-Engineering Center VIM, 1st Institutskiy Proezd, 5, Moscow 109428, Russia; 4 Laboratory of Functional Polymer Materials, National University of Science and Technology “MISIS”, Leninskii prosp, 4, Moscow 119049, Russia; 5 Center for Project Activities, Moscow Polytechnic University, Bolshaya Semenovskaya st., 2, Moscow 107023, Russia; 6 Moscow Aviation Institute, Volokolamsk sh., 4, Moscow 125993, Russia

**Keywords:** polymer composites, nonwoven fabrics, poly-3-hydroxybutyrate, polylactide, polymer substrate, germination, wheat seeds

## Abstract

Electrospun fabrics have unique properties due to their uniform morphology and high surface area to volume ratio. Ultrathin nonwoven fabrics are produced for many applications: biomedical, nanosensors, tissue engineering and filtration systems. In this work, nonwoven polylactide, polylactide/natural rubber, poly-3-hydroxybutyrate, and poly-3-hydroxybutyrate/nitrile butadiene rubber fabrics were prepared by electrospinning methods. The obtained fabric samples were used as substrates for the growth of winter wheat seeds “Yubileinaya 100” (*Triticum aestivum L*.). The stimulating effect of polymer substrates on seed germination and plant growth was shown. The structure and properties of nonwoven agromaterials were controlled by differential scanning calorimetry, IR-spectroscopy, and optical microscopy. The mechanical properties of the obtained fabrics before and after their utilization as substrates were studied. After the wheat growing experiment, the degree of crystallinity of PHB and PHB/NBR samples decreased by 12% and they completely lost their mechanical properties. It is shown that the main factors providing the efficiency of seed growth technology on polymer substrates are the chemical nature and structure of the biodegradable matrix.

## 1. Introduction

Currently, in the agricultural industry, much attention is paid to technologies that use biodegradable polymeric materials for the creation of environmentally friendly products and other applications [1,2]. For example, there are technologies that use films made of polymer compositions based on cellulose, starch, and other natural biopolymers for mulching the soil and making pellets and ribbons for vegetable seeds that are planted in the ground using precision farming technology [3,4].

Most materials for agriculture are made from petroleum-based polymers [5]. To reduce the consumption of petroleum products, as well as the environmental impact associated with their consumption, it has recently been proposed to use polymers based on renewable resources to make mulch films and nonwovens for growing crops.

To impart degradation properties, for example, to a polyethylene mulch film, it is possible to use special additives, such as photodegradable substances and oxidants [6], but the biodegradability of polyethylene with additives for photodegradation is insufficient [7]. The new classification of biodegradable materials excludes oxo-degradable and photodegradable mulches from polyethylene and polypropylene [8]. The definition of a new biodegradable material assumes that it is a material that can be destroyed by the action of microorganisms (bacteria, fungi, or algae). The products of this degradation are water, carbon dioxide, or methane (only under anaerobic conditions), and possible residues and new biomass do not harm the environment [8,9].

Of the available range of bio-based polymers, polylactide (PLA) and poly-3-hydroxybutyrate (PHB) are key candidates for agro-industrial applications. The advantage of these materials is their biodegradability, which potentially solves the growing problem of environmental pollution from plastic waste [10].

Polylactide-based compositions are used for pre-sowing seed treatment, as well as to produce covering and mulching materials [9]. Recently, the technology of planting grain seeds in a ribbon has also been developed [11,12]. Such materials will not only provide seeds with protection from the effects of aggressive environmental factors but will also create the necessary microclimate for favorable germination of seeds and plant development. The experience in the successful use of biodegradable materials such as PHB, PLA, and mixtures based on them in various industries is described in the literature [13,14,15,16].

As it is known, to start seed germination, water, carbon dioxide, and heat are required. In this case, it is obvious that the initial stage of seed germination on a polymer substrate is primarily provided by the rate of water supply. For this purpose, the polymer substrate must provide water access to the caryopsis and its embryo in contact with it.

The substrate material must have a corresponding value for the diffusion coefficient of water. The high rate of water diffusion to the seed embryo is ensured by the free volume in the polymer matrix, especially in the area directly contacting the grain. Correspondingly, it can be concluded that the first factor controlling the effectiveness of the stimulating action of the substrate is its structure, which provides a high diffusion flow of water to the seed. It is obvious that a loose structure of nonwoven fibers can provide a high rate of water diffusion to the embryo, which affects the germination rate of the seeds in contact with it.

Despite the many positive properties of biodegradable polymers, their use in agriculture is hampered by their cost and lack of research in this area. The issue of the price of PLA and PHB may be overshadowed by the ecological effect and the importance of producing healthy agricultural products. Probably, the application of bio-based polymer substrates containing natural stimulating and antimicrobial components would reduce the number of pesticides used [17].

PLA and PHB are linear aliphatic biodegradable polyesters obtained from waste vegetable raw materials [18,19]. It is known that both PLA and PHB are very brittle polymers [20,21]. To increase their elasticity, various types of rubber have been added [22,23]. The authors of [22] improved the compatibility of the components of the PLA/NR blends by adding dicumyl peroxide (DCP). At 3% DCP, a significant increase in impact strength and elongation at break was shown. In [23], nanocomposites based on blends of PLA, acrylonitrile butadiene rubber, and 4 wt% organically modified nanoclay were prepared by melt compounding. All nanocomposites exhibited a significant increase in elongation at break. 

In our study, natural rubber (NR) has been used for the PLA modification, and nitrile butadiene rubber (NBR) has been added to the PHB matrix. Nonwoven fibrous materials PLA, PLA/NR, PHB, and PHB/NBR were obtained by electrospinning, which is an economical, efficient, and versatile method for manufacturing fiber materials with nanofiber and submicron fiber diameters. Electrospinning technology is increasingly interesting as a technology for processing biodegradable polymers [24,25]. Polymer fabrics prepared using the electrospinning method have unique properties that depend on electrospinning parameters [25]. The formation and properties of nonwoven fabrics from PLA and PHB composites are discussed by the authors of [26,27]. The effect of annealing in water was also studied [28]. An increase in the time of exposure of the polymer in aqueous medium at elevated temperature (45 and 70 °C) results in a reduction in the radical concentration and the correlation time. In [29,30], the agricultural application of PLA-based nonwoven fibers was considered. In [30], PLA/NR electrospun fabrics were used as substrates for growing basil. The influence of soil exposure on the morphology, thermal, and structural-dynamic characteristics of polymer samples was studied.

The first step of the present work was to obtain bio-based fabrics and study their morphology and properties. Changes in the structure were recorded by spectral, thermal, and optical methods. Additionally, the mechanical characteristics were investigated. Then, it was necessary to evaluate the possibility of using nonwoven fabrics based on biodegradable PHB and PLA as substrates for seed germination, to investigate the change in the structure and properties of the fibrous materials before and after their use as seed mats and to evaluate the most promising agricultural material.

## 2. Materials and Methods

### 2.1. Sample Preparation

For seed substrates, materials made of polymers were used: poly-3-hydroxybutyrate (P-3HB, PHB throughout the text) in powder form produced by the company “Biomer” (Germany) with a molecular weight of Mw = 2.5 × 10^5^ g/mol and a density of 1.248 g/cm^3^, polylactide (PLA) grade 4032D produced by the company “Nature Works” (USA) with a molecular weight of Mw = 1.7 × 10^5^ g/mol and a density of 1.24 g/cm^3^. Natural rubber (NR) SVR-3L with a Mooney viscosity of 50 ± 5 (100 °C) and a poly(cis-1,4-isoprene) content of 91–96 wt.% was kindly supplied by Vietnam Rubber Group (Ho Chi Minh City, Vietnam) and nitrile butadiene rubber (NBR) with 28 wt% nitrile groups was produced by Sibur LLC (Russia). The PHB/NBR and PLA/NR mixtures contained 15 wt% of rubbers. Chloroform was used as a solvent.

Nonwoven fabric was prepared by electrospinning by exposure to an electric voltage of 18–25 kV on an electrically charged jet of a 9 wt.% polymer solution flowing from a capillary nozzle. The consumption of the solution was in the range of (9–11) × 10^−5^ g/s. The distance between the needle tip and the collector was 17 ± 1 cm.

**Figure 1:**
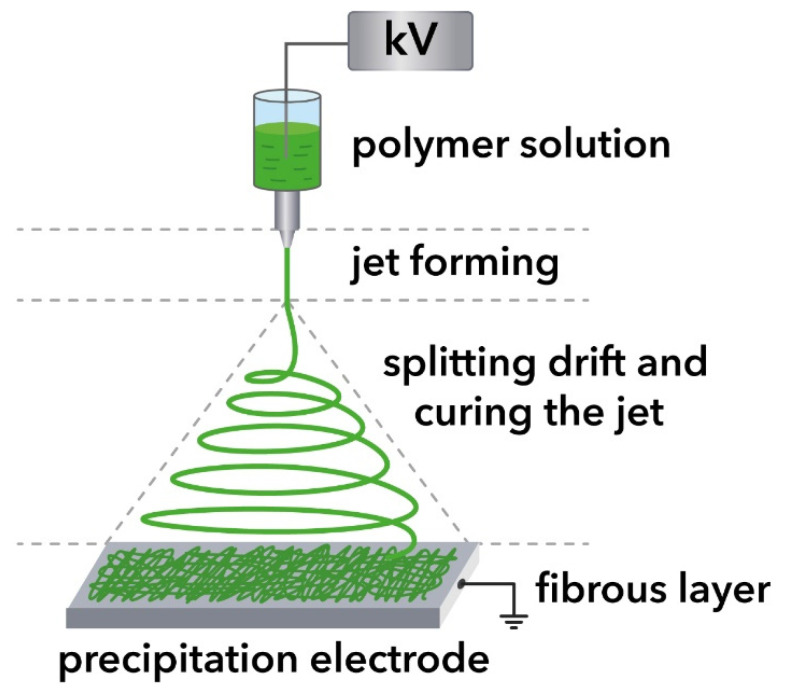
Fabrication of electrospinning of fibers.

The scheme of electrospinning is shown in Figure 1.

### 2.2. Seed Germination Test

The objects of the study were the seeds of the winter wheat variety “Yubileinaya 100” (*Triticum aestivum L.*) harvested in 2019 (Krasnodar Territory). Seed germination, growth, and development of wheat seedlings were studied in laboratory conditions. To germinate the seeds, they were placed in Petri dishes of 100 seeds each in accordance with GOST 12038-84 [31] on the substrates in such a way that in the future they were filled with distilled water, which covered the seeds completely. The temperature of the experiment was 25 ± 0.5 °C. The experiment was carried out using a thermostat while maintaining a constant humidity level. As a standard for monitoring seed germination and plant development on polymer samples, a substrate of ash-free paper filters of the Blue Ribbon FM brand without admixture according to TU 2642-001-68085491-2011 was used.

### 2.3. Analysis of Crystallization

The parameters of polymer substrates were analyzed. The thermophysical characteristics (melting and crystallization temperatures, melting enthalpy) of polymer materials were determined using a differential scanning calorimeter DSC 214 Polyma Netzsch (Germany) at a heating rate of 10 degrees/min in the temperature range of 30–200 °C in the argon stream. The weighted part of the sample varied in the range of 5.5–7.5 mg. The temperature measurement accuracy was 0.1 °C. The melting temperatures were determined by the endothermic maximum of the melting peak on DSC thermograms. The root-mean-square deviations of the experimental areas of the melting peaks of different samples (at least 5 samples) were within 10%. The degree of crystallinity χ_c_ was calculated according to Formula (1):

χ_c_ = (ΔH/ΔH_m_*) × 100, (1)

where ΔH_m_*—the enthalpy assuming 100% crystalline PLA homopolymer 93 J/g [32,33] and PHB homopolymer 146 J/g [33].

### 2.4. Morphology of the Sample

The non-woven fabric’s morphology was examined using an Olympus BX3M-PSLED (Japan) optical microscope of 50×, 200× in reflected light.

### 2.5. Testing of Mechanical Properties

The mechanical properties were examined by a tensile compression testing machine, Devotrans DVT GP UG 5 (Turkey), at a rate of 50 mm/min according to ISO 527-1:2012. The number of samples of each composition was 7. The extreme values were excluded from the results. The tensile strength and elongation at break were determined.

### 2.6. Water Uptake

The parameters of water sorption by the nonwoven material of seed carriers were determined in accordance with GOST 4650-2014 (ISO 62: 2008) [34]. For testing, we used square samples with a side of 30 mm. The test was performed on five samples of each composition. Before testing, the samples were dried at (40 ± 2) °C for 24 h, and then cooled in a desiccator at (22 ± 2) °C and weighed no more than 5 min after being removed from the desiccator. Further, the samples were placed in a vessel with distilled water and an amylase extract was taken in an amount of at least 8 cm^3^ per 1 cm^2^ of the sample surface. The test samples must not be in contact with each other, as well as with the walls of the vessel, and must be completely covered with water. When determining the maximum water absorption to a state of equilibrium, the equilibrium was considered achieved if the difference between the mass of the samples determined with an interval of 24 h did not exceed 0.1%.

After reaching an equilibrium amount of water in the sample, it was removed from the water, dried with filter paper, and weighed on an electronic scale in no more than 1 min. The degree of water absorption was calculated as (2):

(2) $$W=\frac{\left(m_{2}-m_{1}\right)}{m_{1}}\times 100$$

where *m*_1_—initial mass of the sample, *m*_2_—mass of the sample after exposure to water.

### 2.7. FTIR-ATR Spectroscopy

The infrared spectra of the samples were recorded using a Lumos Bruker FTIR spectrometer (Bruker Corp., Bremen, Germany) at T = (23 ± 2) °C in transmitted light in the range of wave numbers 4000 ≤ ν ≤ 600 cm^−1^. For the mathematical processing of the data, the ACD labs software was used.

## 3. Results and Discussion

**Figure 2:**
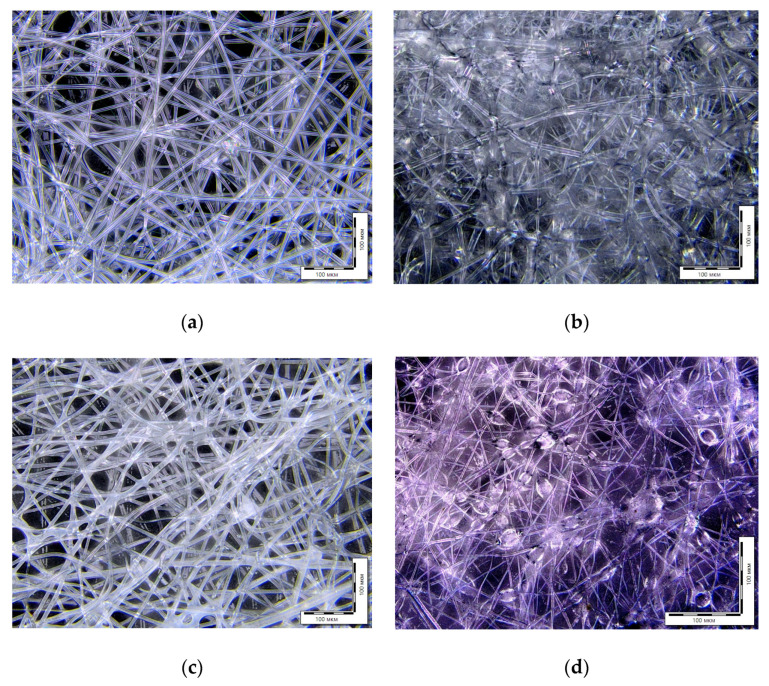
Optic micrographs of nonwoven fabric samples PLA (**a**), 85PLA/15NR (**b**), PHB (**c**), and 85PHB/15NBR (**d**).

The morphology of the obtained samples was studied by optical microscopy (Figure 2). There is a difference between the structure of 100% polymers (PHB and PLA) and their composites (PHB/NBR and PLA/NR). PLA and PHB fabrics have even contours of a single fiber without significant defects and thickenings. PHB/NBR and PLA/NR electrospun composites have a different morphology: a beaded structure is observed. The formation of “beads” is due to the difference in viscosity between rubber and polyester. Since both systems belong to the “thermoplastic/rubber” type, the morphological features of these composites are similar.

**Figure 3:**
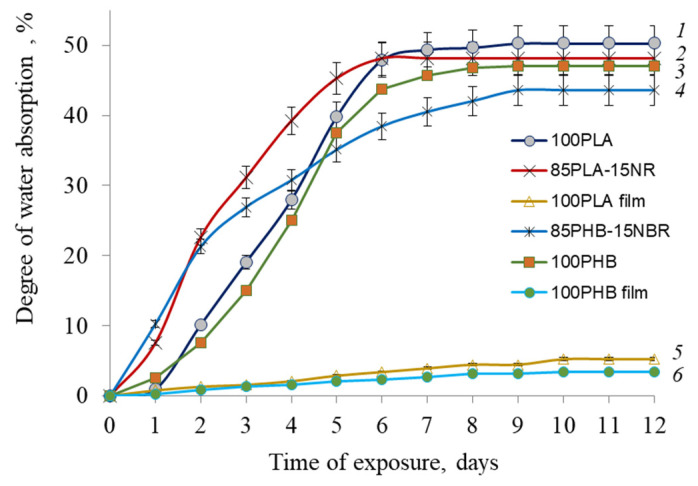
Water uptake of nonwoven fabrics: PLA (1), 90PLA/15NR (2), PHB (3), 90PHB/15NBR (4), and films: PLA (5) and PHB (6).

Even though PLA and PHB are hydrophobic polymers, the degree of water absorption by nonwoven materials is much higher than that of film materials, which is an important factor for seed germination on agricultural material (Figure 3).

The degree of water absorption of film materials does not exceed 3–5%, which indicates the hydrophobic nature of polymers and poor diffusion of water into PLA and PHB films. At the same time, the sorption capacity of nonwoven materials turned out to be much higher and amounted to 43–49%. The addition of NR and NBR slightly reduces this indicator, which may be due to the peculiarities of the morphology formed in the PLA/NR and PHB/NBR composites, which somewhat prevents the absorption of water. This feature is confirmed in other works [29].

**Figure 4:**
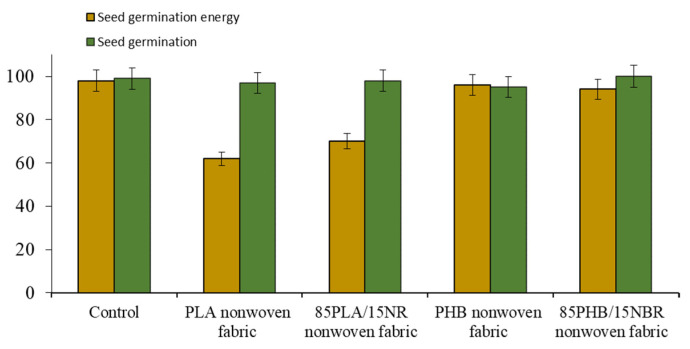
The seed germination energy and the germination of the wheat Yubileinaya 100.

Then, an assessment of seed germination on PLA, PLA/NR and PHB, PHB/NBR nonwoven fabrics was carried out. There is a tendency to increase the germination energy of seeds and a noticeable increase in the parameters of the root system and seedlings of plants compared with the control substrate (Figure 4) [35,36].

**Figure 5:**
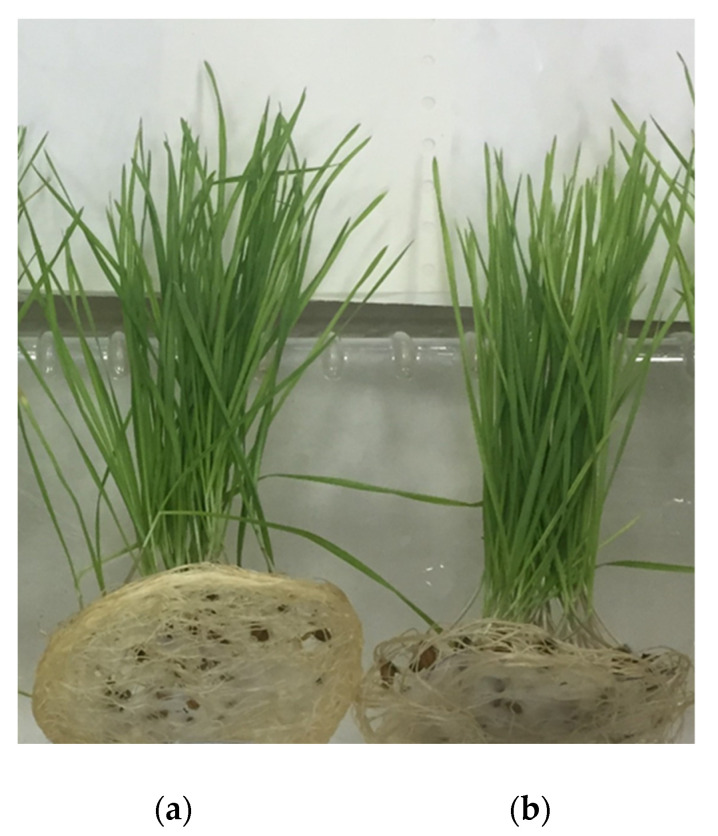
Photographs of PHB (**a**) and 85PHB/15NBR (**b**) nonwoven samples after a germination of wheat plants on the 12th day of vegetation.

According to the experimental data, the values of the germination energy of the germination of wheat seeds on polymeric substrates are close to the control values. Thus, the use of polymeric substrates does not have a significant effect on germination. However, fibrous substrates affect the biometric indicators of wheat plants. Wheat plants were carefully removed from the polymeric substrates and measured. Since the laboratory experiment is carried out up to the stage of germination and development of 1, 2, and 3 leaves, the biometric indicators were determined on the 12th day of vegetation. Images of wheat plants germinated on PHB and PHB/NBR nonwoven fabrics are shown in Figure 5.

Analysis of the data in Table 1 showed that the biometric characteristics of the structural elements of wheat seedlings in the control sample are significantly lower than those of seedlings grown on polymeric substrates of all types.

The height of wheat plants has similar values for all polymeric substrates. On average, this indicator is 1 cm higher than the control value. The mass and length of the roots are significantly greater than in the control sample. The plant weight is also higher in plants growing on nonwoven fabrics.

For grain at the stage of ontogenesis, the determining factor is the rate of swelling of its embryo in an aqueous medium [37,38]. One of the fundamental factors that affects the germination of plants is water, which should be sufficiently supplied through a polymer seed carrier. This is achieved not only by the diffusion flow of water, but also by the swelling of the polymer in contact with the caryopsis. The germination of seeds leads to the beginning of the degradation of polymer substrates. The active medium destructively acting on the polymer matrix of the substrate is the enzymes.

On the example of polylactide, we can note that the hydrolytic degradation of PLA-based materials occurs by breaking the ester bonds, while the mechanism of hydrolysis depends significantly on the pH of the medium in which the process is realized [39,40]. The degradation process begins with the nucleophilic attack of the terminal hydroxyl of another carbonyl group. This process results in the formation of lactic acid oligomers and lactide, which is degraded to lactic acid. In an acidic environment, the degradation of polylactide is initiated by protonation of the end ester group followed by the formation of an intramolecular hydrogen bridge. The products of hydrolytic degradation by this mechanism are lactic acid and lactic acid oligomers [40]. Chain cleavage reaction during hydrolytic degradation of PLA proceeds mainly in amorphous regions, which leads to an increase in the crystallinity of the polymer [41].

**Figure 6:**
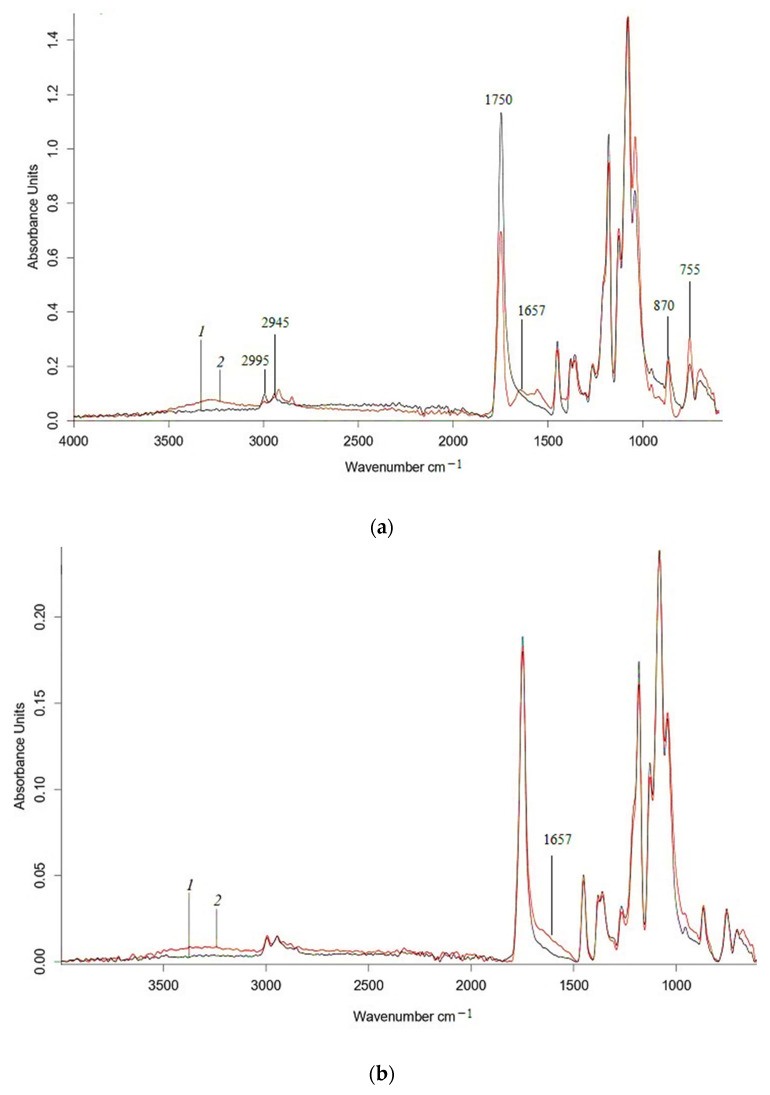
ATR-IR spectra of PLA (**a**), 85PLA/15NR (**b**) nonwoven fabrics (1) and 60 days after water (2).

**Figure 7:**
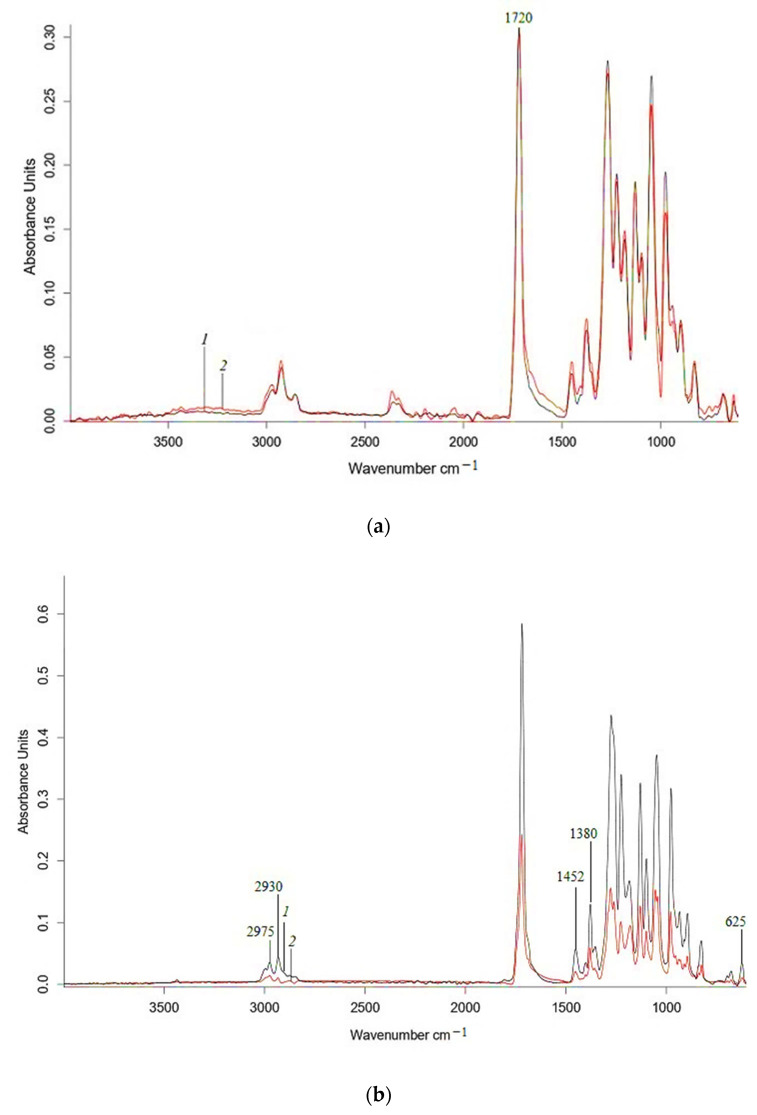
ATR-IR spectra of PHB (**a**) and 85PHB/15NBR (**b**) nonwoven fabrics (1) and 60 days after water (2).

Analyzing the effect of water on nonwoven PLA, PLA/NR and PHB, PHB/NBR agromaterials using the ATR-IR method, it should be noted that the materials are subject to hydrolytic degradation (Figure 6 and Figure 7).

The IR spectra (Figure 6) show changes in the characteristic polyester bands, which are subject to hydrolytic degradation. In Figure 6 and Figure 7, in the region of 1300–980 cm^−1^, there is a decrease in the fluctuations of ester groups. Similar changes are present in the range of vibration of –C=O groups (1750 cm^–1^), which confirm the occurrence of the hydrolysis process in the polylactide matrix, as well as visible changes in the structure-sensitive bands at 755 and 865 cm^–1^, which refer to –C–C– vibrations of the crystalline and amorphous phases of polylactide, respectively [42]. The intensity of the characteristic PHB band at 625 cm^−1^, which refers to vibrations of –C–C– bonds in methylene sequences, also decreases after exposure to water. The IR spectra of the PLA, PHB, and their composites exhibit two distinct peaks at 2995 and 2945 cm^–1^, which correspond to asymmetric and symmetric vibrations of the –CH_3_ group, respectively. In the region of 3500–3000 cm^−1^, an increase in intensity occurs, which may correspond to hydroperoxides (Figure 6 and Figure 7).

Not only polymer substrates affect the germination and growth of wheat but plants also affect the process of polymer matrix destruction. Enzymatic hydrolysis of the polymer substrate triggers the stage of disintegration of PLA and PHB matrixes and the development of biochemical processes. The degradation rate of a polymer depends on its nature and structure. Germination of seeds, their development and growth of seedlings are accelerated depending on the rate of decomposition of the polymer mass.

As mentioned above, hydrolytic processes lead to a violation of the structure of the material. These changes are reflected in the thermophysical characteristics of PHB and PLA materials. In the sample subjected to hydrolysis, the structure of the crystalline regions changes. In a sample subjected to enzymatic hydrolysis, the decrease in values of melting temperatures (T_m_) reaches 4 °C for PLA fabrics and 10 °C for the PHB. The degree of crystallinity changes by 12% in PHB and PHB/NBR agromaterials (Table 2).

**Figure 8:**
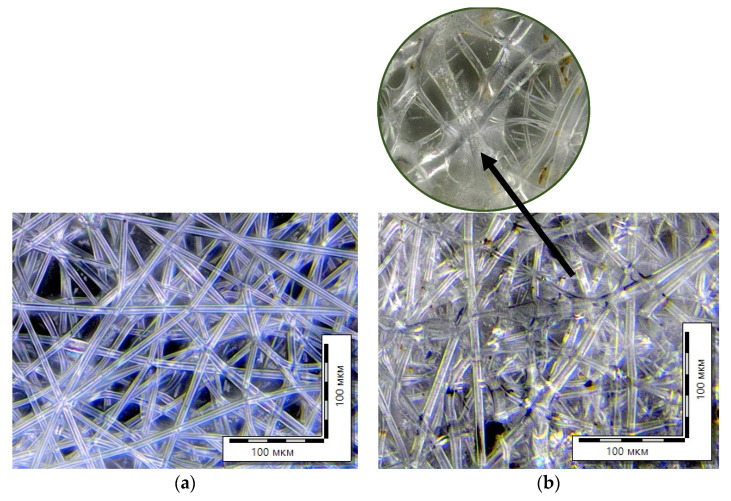
Optical micrographs of nonwoven samples: initial PLA (**a**) and PLA substrate (**b**).

It is known that the rate of hydrolysis is different for materials with various chemical and physical structures and depends on the presence of crystalline regions in the polymer, access to which is difficult. Hydrolysis can occur both on the surface of the polymer material and in its bulk. Figure 8 shows micrographs of the initial PLA sample and PLA fiber substrates after exposure to water and enzymes during the growth of wheat plants. Clear changes in the structure of the agromaterials are visible. Single fibers seem to blur and become thinner. Spots and defects appear in the morphology, which are the evidence of the degradation process.

**Figure 9:**
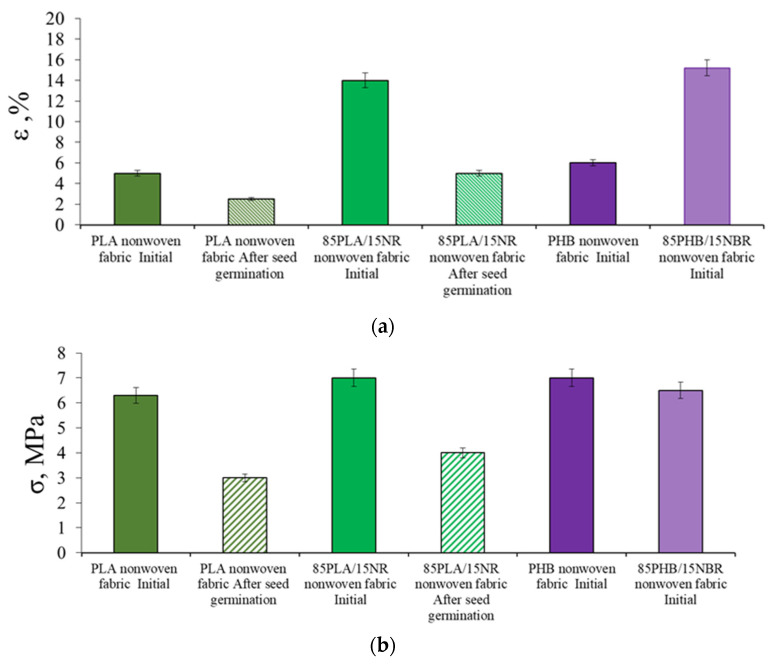
The change in the elongation at break (**a**) and tensile strength (**b**) of PLA, PLA/NR and PHB, PHB/NBR nonwoven fabrics.

The development of the root system on polymer carriers causes changes in their physical and mechanical properties. A study of the tensile strength and elongation at break of PHB, PHB/NBR and PLA, PLA/NR nonwoven fabrics before and after plant contact tests showed that after germination of seeds the values of elongation at break and tensile strength significantly decrease (Figure 9).

After the experiment with wheat germination for PHB and PHB/NBR agromaterials, it was not possible to determine the elongation at break and tensile strength values due to the destruction of nonwoven materials. PLA and PLA/NR composites were found to be more resistant to water and enzymes.

The crystalline regions of PLA are resistive to hydrolysis, which causes selective hydrolytic cleavage of chains in amorphous regions and removal of water-soluble oligomers and monomers formed as a result of hydrolysis, with only some residual crystalline regions [43]. Polymers obtained from natural raw materials, which especially include PHB and PLA, are most susceptible to decomposition. At the same time, when decomposing mixed compositions, decomposition begins with a natural component that completely decomposes and thereby destroys the entire material. If the composite contains a synthetic matrix, then the question arises about the synthetic residue after the decomposition of the biodegradable polymer in the system [44].

As can be seen from the presented data, the polymer fabric is not only the substrate but also the nutrient medium. It is a supplier of carbon dioxide and heat to seeds, which are the products of the biodegradation of polymers that accelerate seed germination. Decomposition products can replace those mineral additives that are introduced into the soil for plant nutrition. The use of biodegradable polymers makes it possible to isolate seeds from the effects of harmful contaminants in the soil. The use of this technology can facilitate the dosed supply of minerals, especially in granulation technology. Seeds can be placed in granules together with the estimated number of necessary fertilizers. Biodegradable polymers can be a good alternative to traditional crop-growing methods. As previously shown, some of them have only a small effect on soil quality over 18 months [45,46].

The biodegradable materials considered in this work, when used as polymer sub-strates in a closed ground, are destroyed in 4–6 months. The roots of developing plants contribute to this destruction. Biodegradable polymers as an encapsulation matrix/coating for controlled-release fertilizers or seeds can be considered an environmentally friendly solution against polymer pollution. Nevertheless, since these polymers still have a higher cost compared to traditional polymers, the introduction of new environmentally friendly materials in agriculture is slow. However, the importance of addressing environmental aspects is relevant.

## 4. Conclusions

Agromaterials based on PLA and PHB were obtained by electrospinning. Laboratory experience in growing Yubileinaya 100 wheat showed differences both in biometric parameters and germination for the control sample and polymer substrates. It was found that on the 12th day of wheat vegetation, the degree of degradation of PHB and PHB/NBR polymer substrates is higher compared to PLA and PLA/NR fabrics. The samples of PHB and PHB/NBR show a complete loss of mechanical properties.

Thus, the data obtained demonstrate that nonwoven fabrics are promising for agriculture applications. The using of polymer fabrics can be a new step in the field of crop and seed production. Polymer fabrics are not only the substrates but also the nutrient medium. The rate of decomposition of PLA, PLA/NR and PHB, PHB/NBR nonwoven fabrics, which determine the stimulating ability in relation to plant seeds, depends on their structural features, their ability to swell and their disintegration in an aqueous solution of enzymes, and their activity to oxidative degradation.

The current study suggests that fabrics based on biodegradable polyesters can be used as covering materials to protect the seedlings of expensive elite crop varieties from birds and wind. However, for the application of new methods of growing agricultural crops, a number of studies need to be carried out. It is necessary to conduct field experiments, evaluate the features of growing crops in open ground, and study the effect of aggressive environments on polymer fabrics.

## Figures and Tables

**Table 1:** Biometric characteristics of wheat plants of the Yubileinaya 100 variety on the 12th day of vegetation.

Type of the Substrate Sample	Weight, g		Root Length, cm	Plant Height, cm
	1 Plant	Roots		
Control sample	0.156 ± 0.018	0.035 ± 0.004	8.8 ± 0.7	11.4 ± 0.4
PLA	0.184 ± 0.023	0.054 ± 0.005	11.0 ± 0.8	12.0 ± 0.5
85PLA/15NR	0.191 ± 0.022	0.052 ± 0.003	12.3 ± 0.6	12.6 ± 0.5
PHB	0.182 ± 0.019	0.052 ± 0.004	13.4 ± 0.6	12.8 ± 0.4
85PHB/15NBR	0.196 ± 0.020	0.050 ± 0.003	10.8 ± 0.7	12.5 ± 0.3

**Table 2:** Thermophysical characteristics of PLA, PHB, and their fiber composites.

Type of the Substrate Sample	T_m_, °C (Δ ± 0.3 °C)	ΔH_m_, J/g (Δ ± 0.5 °C)	χ_c_, % (Δ ± 0.5%)
PLA nonwoven fabric (*n*/w) initial	165	37	40
PLA *n*/w after seed germination	161	35	38
PLA *n*/w after saturation with water	166	43	46
85PLA/15NR nonwoven fabric (*n*/w) initial	166	36	38
85PLA/15NR *n*/w after seed germination	165	32	34
85PLA/15NR *n*/w after saturation with water	168	42	45
PHB nonwoven fabric (*n*/w) initial	173	84	58
PHB *n*/w after seed germination	163	53	36
PHB *n*/w after saturation with water	171	82	56
85PHB/15NBR nonwoven fabric (*n*/w) initial	170	76	52
85PHB/15NBR *n*/w after seed germination	163	59	40
85PHB/15NBR *n*/w after saturation with water	165	60	41

## Data Availability

The data presented in this study are available on request from the corresponding author.
